# Using Doubly-Labeled Water to Measure Energy Expenditure in an Important Small Ectotherm *Drosophila melanogaster*

**DOI:** 10.1016/j.jgg.2014.07.004

**Published:** 2014-09-20

**Authors:** Matthew D.W. Piper, Colin Selman, John R. Speakman, Linda Partridge

**Affiliations:** a Institute of Healthy Ageing, Department of Genetics, Evolution and Environment, University College London, Gower Street, London, WC1E 6BT, UK; b Institute of Biodiversity Animal Health and Comparative Medicine, University of Glasgow, Glasgow, G12 8QQ, UK; c Institute of Biological and Environmental Sciences, University of Aberdeen, Aberdeen, AB24 2TZ, UK; d State Key Laboratory of Molecular Developmental Biology, Institute of Genetics and Developmental Biology, Chinese Academy of Sciences, Beijing, 100101, China

**Keywords:** Doubly-labeled water, Respiration rate, Effect of mating status and sex, *Drosophila*

## Abstract

Energy expenditure is a key variable in the study of ageing, and the fruit fly *Drosophila melanogaster* is a model organism that has been used to make step changes in our understanding of the ageing process. Standard methods for measurement of energy expenditure involve placing individuals in metabolic chambers where their oxygen consumption and CO_2_ production can be quantified. These measurements require separating individuals from any social context, and may only poorly reflect the environment in which the animals normally live. The doubly-labeled water (DLW) method is an isotope-based technique for measuring energy expenditure which overcomes these problems. However, technical challenges mean that the smallest animals this method has been previously applied to weighed 50–200 mg. We overcame these technical challenges to measure energy demands in *Drosophila* weighing 0.78 mg. Mass-specific energy expenditure varied between 43 and 65 mW·g^−1^. These estimates are considerably higher than estimates using indirect calorimetry of *Drosophila* in small metabolic chambers (around 18 mW·g^−1^). The methodology we have established extends downwards by three orders of magnitude the size of animals that can be measured using DLW. This approach may be of considerable value in future ageing research attempting to understand the genetic and genomic basis of ageing.

## Introduction

Energy has long been regarded as a limiting resource that can mediate trade-offs between different life-history activities, such as reproduction and survival, and hence is of central importance in the ageing process (Williams, 1966; Kirkwood, 1977). Indeed the earliest and most enduring theory of ageing was based on the idea that ageing is driven directly by levels of energy demand (Pearl, 1928). Although our understanding of the ageing process and how it is linked to energy expenditure has moved on from these simplistic model (Speakman et al., 2002; Speakman, 2005), evaluating exactly how organisms use energy under different circumstances, for instance by the two sexes during reproduction, remains fundamental to understanding diversity in life histories and the constraints under which they evolve (Stearns, 1992; Speakman, 2008). Methods to accurately quantify total energy expenditure in organisms as they engage in a variety of life-history pursuits are therefore essential.

Direct calorimetry uses accurate measurement of heat production to determine energy expenditure. This technique is experimentally challenging, particularly if the organism is small, and it is also expensive (Levine, 2005). Alternatively, quantification of energy expenditure can be made by measurement of respiratory gas exchange (indirect calorimetry). The classical open-circuit indirect calorimetry method requires a subject to be placed within a sealed chamber with a continuous airflow, and then the exhaled respiratory gases are analyzed (Selman et al., 2001; Levine, 2005). One disadvantage of this method is that there is a requirement to confine the subject within a sealed metabolic chamber, and ultimately this may restrict the natural levels of activity. Secondly, it is impossible using this method to discern the metabolism of focal individuals within a social setting – for example a mixed-sex group.

**Fig. 1:**
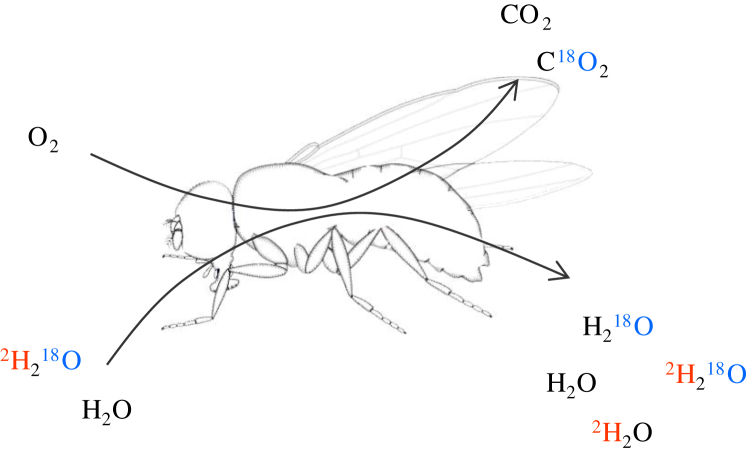
Illustration of isotope uptake and elimination when using doubly-labeled water to measure metabolic rate. ^18^Oxygen and ^2^Hydrogen are artificially enriched in the body water pool and their subsequent elimination was measured. This occurs at different rates for the two isotopes because hydrogen is primarily eliminated by the outflow of body water, while oxygen is eliminated both by water loss and from expiration of respiratory CO_2_. The difference between the elimination rates of the two isotopes therefore provides an estimation of CO_2_ production in an individual over a given time.

The doubly-labeled water (DLW) technique (Lifson et al., 1955; Speakman, 1997; Nagy, 1983) is an isotope-based method that can overcome some of the issues highlighted above. DLW involves labeling the body with isotopes of oxygen and hydrogen (normally ^18^Oxygen (^18^O) and ^2^Hydrogen (^2^H), which are stable non-radioactive isotopes). The elimination of these rare isotopes occurs from the body water pool at different rates, because the hydrogen label is eliminated primarily *via* outflow of water from the body water pool, while the oxygen label is eliminated both by water loss and from expiration of respiratory CO_2_ (Fig. 1). The difference between the elimination rates of these two isotopes therefore provides an estimation of CO_2_ production and hence respiration, in an individual over a known time period.

Historically, the DLW method has primarily been applied to animals with high metabolic rates, because this facilitates a strong divergence between the ^18^O and ^2^H elimination curves (Speakman et al., 1993; Berteaux et al., 1996). Sensitivity analyses have shown that the magnitude of the divergence in the elimination curves is the principal driver of precision in the method (Speakman, 1995). Therefore, the majority of DLW studies have involved birds, reptiles and mammals (reviewed in (Nagy et al., 1999; Speakman, 2000; Anderson et al., 2003)), including humans (Schoeller, 1988; Goran et al., 1994; Speakman et al., 2002; Westerterp and Speakman, 2008). While several measurements have been made on arthropods with a body mass (BM) greater than ∼250 mg (scorpions (King and Hadley, 1979); tenebrionid beetles (Cooper, 1983); bumble bees (Wolf et al., 1996)), in animals with a BM ∼50 mg or less, size starts to become a challenge both in terms of dosing and for evaluation of elimination rates. This is because the animals are too small to inject with weighed quantities of the isotopes. Furthermore, their mass-specific metabolic rate may be so high that the sampling period is difficult to discern due to rapid washout and, finally, because they do not provide a sufficiently large sample of body water for analysis by isotope ratio mass spectrometry (Butler et al., 2004). These limitations have so far precluded the use of this DLW method for key model organisms such as the nematode worm *Caenorhabditis elegans* and the fruit fly *Drosophila melanogaster*.

In the present study, we employed the DLW methodology to measure total energy expenditure in *Drosophila.* This novel approach thereby extended downwards by three orders of magnitude the size of animal to which this method can be applied. We have demonstrated the method by measuring energy expenditure of both male and female *Drosophila* under different reproductive conditions, including focal individuals within mixed-sex populations.

## Results

Employing DLW technique, we determined total energy expenditure in adult male and adult female *Drosophila* under three distinct experimental regimes: 1) non-mated single-sex population (virgin), 2) allowed to mate for 48 h at the beginning of life and subsequently housed separately in single-sex populations (once-mated), and 3) housed in mixed-sex populations for the duration of experiment (mixed-sex).

**Fig. 2:**
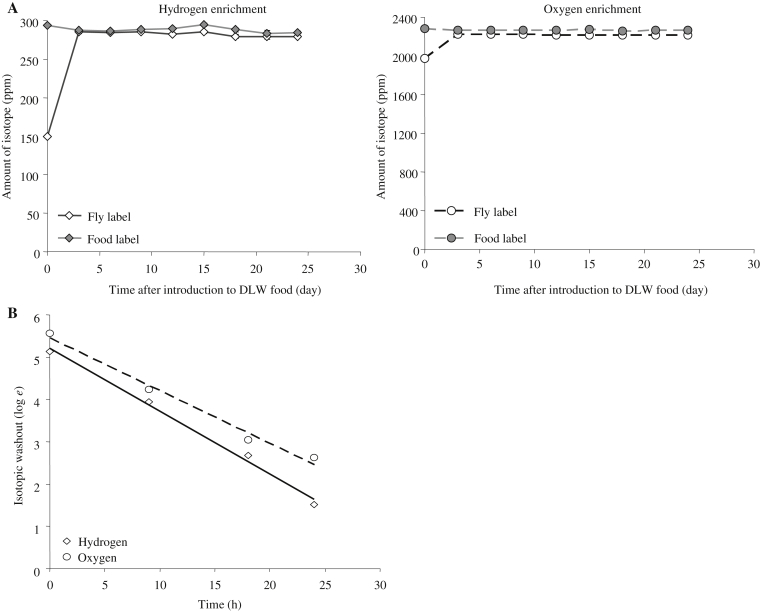
Characteristics of isotope accumulation and elimination from flies. **A:** Enrichment of ^2^Hydrogen and ^18^Oxygen was measured in flies after feeding on DLW-containing food for up to 24 days. Uniform enrichment of label in the flies was observed to occur within 3 days whereupon flies contained close to the same level of isotope enrichment as found in the food. Values plotted are means ± SEM. **B:** Example elimination profile of labeled hydrogen and oxygen. The difference between these exponential elimination rates was used to estimate CO_2_ production.

When flies were fed on isotopically enriched food, their body water isotope enrichment for both ^2^H and ^18^O increased within 72 h to a stable value that closely approximated, but was slightly lower than the corresponding isotope enrichments in the food (Fig. 2A). A typical isotope washout curve, in this case for once-mated females, shows linearity and also divergence of the ^2^H and ^18^O isotopic labels (Fig. 2B). This divergence enabled an estimate of energy expenditure to be derived for each experimental group. The gradient of this decline allowed us to calculate the turnover of the oxygen label (*k*_o_) and the turnover of the hydrogen label (*k*_d_). The *k*_o_, *k*_d_ values and the *k*_o_/*k*_d_ ratio for all subpopulations are shown in Table 1. The mean *k*_o_/*k*_d_ ratio was 1.16. The half-life for ^18^O elimination was 9.2 h and for ^2^H elimination was 10.6 h.

### Measuring respiration rate in flies housed under laboratory conditions

**Fig. 3:**
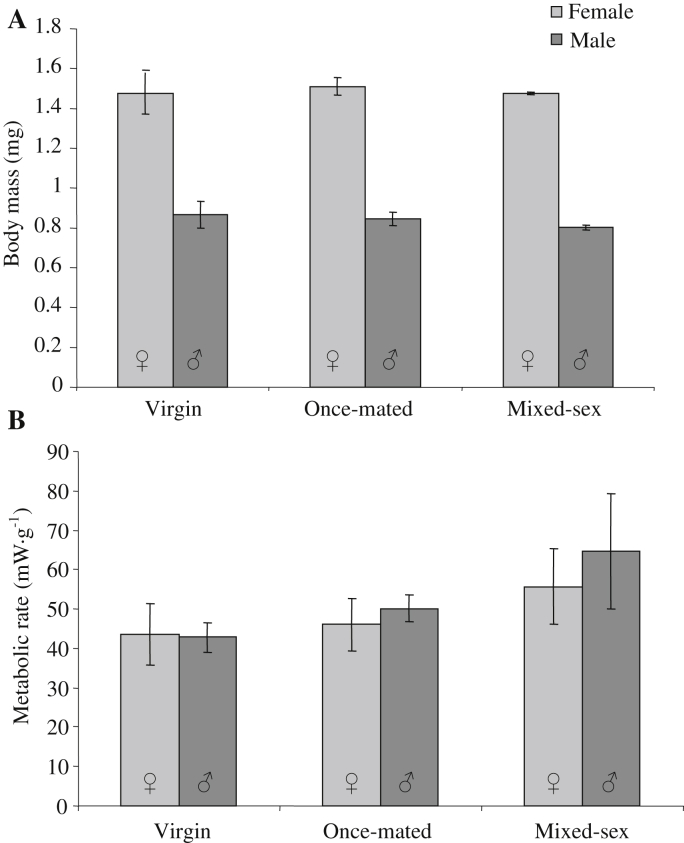
Body weight and metabolic rates of adult flies from each of the experimental cohorts. **A:** The body weight of female flies was significantly greater than male flies, although no treatment effect on BM was observed. **B:** No significant change in metabolic rate was detected for either sex in any treatment condition, although a trend towards an elevated metabolic rate in non-virgin flies was observed. Values are means ± SEM.

A highly significant gender effect on BM was observed, with female flies being significantly heavier (*F* = 700.8, *P* < 0.001; Fig. 3A) compared to males, although no significant treatment (*F* = 0.589, *P* = 0.560) or sex × treatment interaction effect (*P* > 0.05) was seen. Mean mass-specific energy expenditure, expressed as mWatts/gram (mW·g^−1^), was not significantly different between sexes (*F* = 0.376, *P* = 0.543) or between groups (*F* = 2.147, *P* = 0.132) (Fig. 3B), with no significant interaction effect observed (*P* > 0.05). The energy expenditure data were then analyzed in a second way using mWatts and introducing BM as a covariate into a general linear model. Again we observed no significant sex (*F* = 1.037, *P* = 0.315) or treatment effect (*F* = 1.641, *P* = 0.208) on energy expenditure. In addition, no significant BM (*F* = 3.406, *P* = 0.073) or interaction effects (*P* > 0.05) were detected. While there were no significant group effects, in both sexes the lowest metabolic rates were observed in virgin animals and the highest metabolic rates were seen in the mixed-sex groups (Fig. 3B).

By employing a *post hoc* power analysis (80% power, mean standard deviation = 18.7 mW·g^−1^, *n* = 6), we calculated that a difference between groups of 42.3 mW·g^−1^ was required to detect statistical significance (*P* < 0.05). In this study, the maximum difference in energy expenditure was 21.9 mW·g^−1^, between virgin males and mixed-sex males. Therefore, our six replicate studies were slightly underpowered. Nonetheless, the data clearly demonstrate that the DLW technique can be used to measure the energy expenditure of *Drosophila* housed under normal laboratory conditions, and furthermore that it is possible to study the energy expenditure of subpopulations of flies during a period of co-habitation. However, we suggest that future studies using this technique should incorporate a greater degree of replication.

## Discussion

To overcome the technical challenges of dosing the flies, since injection or feeding of a highly enriched isotope bolus was precluded, we fed the animals on food that had been artificially enriched with the two isotopic labels ^2^H and ^18^O. One potential problem with this dietary enrichment, as opposed to these other two approaches, is that the isotope dose is relatively low, and as a result the animals might washout the isotope as rapidly as they take it up. However, our initial analysis demonstrated that when flies were fed on labeled food they rapidly took up the isotopes and achieved a stable equilibrium within 72 h of exposure to the labeled diet (Fig. 2A). This is an important finding as it demonstrates that the labeling of the animals *via* their food source is an effective methodology to determine energy expenditure in small organisms over relatively short-time periods.

The second technical challenge of this study was how to collect body water samples necessary for determining the isotopic enrichments, from an animal weighing ∼1 mg. Assuming a haemolymph volume of approximately 50 nL in *Drosophila* (Folk and Bradley, 2003), taking a sample that removed 10% of the haemolymph would yield a total distillate sample of 5 nL. Currently the smallest samples that can be analyzed by gas source stable isotope ratio mass spectrometry are one to two orders of magnitude greater than this (depending on the machine) (Morrison et al., 2001; Butler et al., 2004) and so this precluded the possibility of analyzing individual flies in our study. We therefore sampled groups of flies (*n* = 50–100) and extracted their entire body water contents for analysis. Because individuals within an experimental group will differ in their energy expenditure, this could interfere with the construction of linear washout curves. However, by using large numbers, i.e., 50–100 individual flies at each time point, we were able to obtain linear washout curves.

The rapid uptake of label we detected in the initial trials (Fig. 2A) suggested that washout of the label would be similarly fast, and this was indeed the case (Fig. 2B). The half-life for elimination of the ^18^O label was only 9.2 h and for ^2^H it was slightly slower at 10.6 h. Nagy (1983) has suggested that 2–3 half lives is the optimal duration for DLW measurements, suggesting that an optimal duration for a study of *Drosophila* is around 20–30 h. Our analysis showed that samples during washout taken at 24 h were already close to the isotopic background, and as a result energy expenditure estimates derived from these 24 h measurements had increased variability. This rapid uptake and elimination of label is also advantageous in that it allows relatively fine temporal resolution of measurements, which is likely to be of use in relatively short-lived organisms such as *Drosophila*.

A variety of methods (Levine, 2005) can be used to measure whole-animal metabolic rate in *Drosophila* and other small ectotherms with their principle advantage over DLW currently being that they can be made on single individuals and so suffer from smaller experimental variation. However, some significant problems with these methods have already been mentioned, including the necessity to confine the subject within a chamber and hence constrain its activity and movement. Studies comparing different methods to determine metabolic rate have often produced conflicting and often idiosyncratic results. It was shown, for example, that manometric methods estimated significantly higher metabolic rates in *D. simulans* compared to those estimated by CO_2_ production using infrared gas analysis (Van Voorhies et al., 2008). However, unlike DLW all these methods do not allow the ability to discriminate daily energy expenditure of focal groups, e.g., males and females, within a social setting. Studies using indirect calorimetry have reported resting metabolic rate (RMR) values for virgin female *Drosophila* at 17.3–19.2 mW·g^−1^ (Hulbert et al., 2004) and 12–16.5 mW·g^−1^ for once-mated male *D. simulans* (Van Voorhies et al., 2008), with data normalized assuming a mean BM of 0.84 mg·fly^−1^). In contrast, our measurements for energy expenditure ranged from 42.7 mW·g^−1^ for virgin males, to 64.6 mW·g^−1^ for males kept in mixed-sex conditions, thus exceeding the RMR values by approximately 3 to 4-fold. The metabolic cost of walking in *Drosophila* is estimated at 5%–10% above RMR (Berrigan and Partridge, 1997) and that for flying is 5–7.4-fold greater than RMR (Dickinson and Lighton, 1995; Laurie-Ahlberg et al., 1985). Thus, under our experimental conditions in which 50 flies were housed with approximately 500 cm^3^ free-space and with free access to excess food, the greater energy expenditure suggests that these animals spend a significant amount of free-time flying. Therefore, it is possible that the restrictive nature under which more conventional assays of metabolism are collected, e.g., indirect calorimetry, is not the best indicator of daily energy requirements, because the metabolic costs of activities which account for a large proportion of the daily energy budget are potentially underestimated.

In summary, we describe a novel protocol that enables the determination of the total energy expenditure budget in *Drosophila* under free-living and socially mixed conditions using DLW technique. This study extends downwards by three orders of magnitude the smallest animals yet measured using this technique (to 0.78 mg), and as a result extends the range of animal body size now studied by DLW to over 9 orders of magnitude, from male *Drosophila* in this study to walruses *Odobenus rosmarus* (Aquarone et al., 2006). These data demonstrate the potential for this technique to address the metabolic costs to *Drosophila* following environmental (e.g., courtship, reproduction, dietary restriction) manipulations. Perhaps of even greater significance would be the potential to evaluate the impact of genetic manipulations that are known to impact lifespan (Partridge et al., 2005; Piper et al., 2008) and to link differences in energy metabolism to genomic profiles.

## Materials and methods

### *Drosophila* rearing and media

The wild-type, outbred Dahomey laboratory stock was used throughout this study. Fly stocks were housed in population cages (20 cm W × 30 cm D × 20 cm H) with overlapping generations, and maintained under a 12 h:12 h/light:dark cycle at 25°C and 65% humidity. For all experiments, flies were reared at standard density for at least two generations (Clancy and Kennington, 2001). A sugar-yeast food medium (SY; for sugar/yeast: 50 g/L sugar (Tate and Lyle Sugars, UK) and 100 g/L lyophilized brewer's yeast (MP Biomedicals, USA)) was used (Bass et al., 2007). For DLW experiments, the ^2^H (Cambridge Isotope Laboratories, USA) and ^18^O (Rotem Industries Ltd., Israel) isotopes were added at 588 μL/L after the food had cooled to 65°C. The labeled food was then stored at 4°C in plastic bags until use.

### Experimental groups

Flies were reared at standard density of approximately 300 flies per 200 mL glass bottle containing 70 mL of SY food. Virgins (referred to as the ‘virgin’ group) were collected under cold anesthesia (ice). The remaining mixed-sex flies were also anesthetized on ice and then transferred, without sorting, to fresh bottles for 48 h. After 48 h, all flies were anzesthetized using CO_2_, counted and transferred to fresh bottles at a density of 50 flies per bottle, either as single-sex groups (referred to as ‘once-mated’) or as mixed-sex groups of 25 females and 25 males (referred to as ‘mixed-sex’). Flies were transferred without anesthesia every third day to fresh bottles until assayed.

### Establishing the time required to label the animals *via* food intake

Two-day-old and once-mated males were transferred to bottles containing 15 mL of labeled food. At the same time, triplicate samples of 100 males maintained on unlabeled food were taken for background isotope measurements of unlabeled flies. Flies on DLW food were transferred every third day to fresh bottles, and on days 3, 6, 9, 12, 15, 18, 21 and 24, triplicate samples of 100 flies were collected, together with triplicate samples of the food from the vacated bottles. Prior to sampling, flies were transferred without anesthesia to empty bottles for 1 h to clear labeled food from the gut, since the gut passage time in *Drosophila* is approximately 50 min (Wong et al., 2008). Flies were then snap frozen in liquid nitrogen and stored at −80°C until analysis.

### Establishing the dynamics of label washout

Groups of once-mated males and females were used. Eight-day-old males and females were either taken as duplicate samples of 100 as unlabeled controls or transferred to DLW food. After 72 h on labeled food, duplicate samples of 100 flies were taken for each sex (washout *t* = 0) and the remaining flies were subsequently maintained on unlabeled food. From these remaining flies, duplicate samples of 100 flies for each sex were taken at 18, 24, 48 and 72 h.

### Determining the energy expenditure for flies of different sex and mating status

**Fig. 4:**
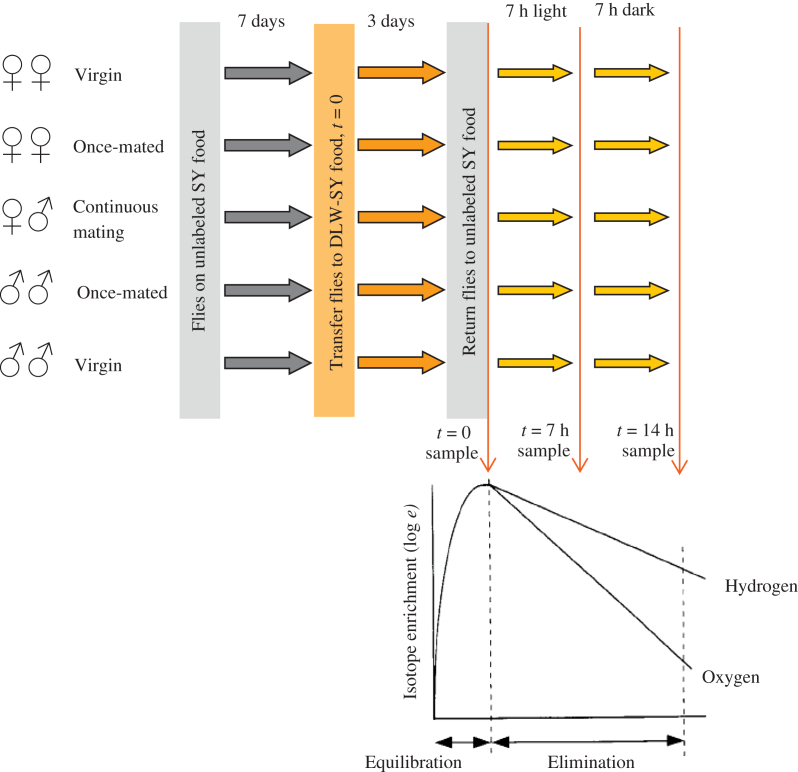
Experimental plan for measuring respiration rate in *Drosophila* after feeding isotopically labeled food. Upon pupariation, flies were sorted into experimental groups and maintained on normal food (represented by grey arrows). After one week, the adults were transferred to food containing DLW (orange arrows). After three days, flies were considered uniformly labeled. A sample was taken (*t* = 0) and the remaining cohort transferred back to unlabeled food (yellow arrows). Over the next 14 h, the remaining samples were taken for isotope analysis to assess the rates of isotope elimination. Because circadian cycles can affect the DLW estimates of energy demands (Speakman and Racey, 1986), we timed the measurement period of 14 h to span equal intervals of 7 h light and 7 h dark.

Cohorts of virgin males and females, once-mated males and females and mixed-sex males and females were established. Flies were treated as described above, with the following exceptions. Upon removal from food, all flies were anesthetized under CO_2_ before transfer to empty bottles. This was to allow male and female flies from the mixed-sex groups to be separated before freezing. In addition, samples in trial 1 (virgin females) were taken at 0, 9, 18, 24 and 36 h and in trial 2 (virgin males), at 0, 6 and 13 h and in trial 3 (once-mated females only) at 0, 7 and 14 h (Fig. 4). To avoid potential energy expenditure differences arising from circadian rhythmicity, we timed all our measurements to ensure that the whole duration of sampling always spanned equal light and dark phases of the daily cycle.

### Isotope analysis and calculations

Sampled flies were weighed (±0.0001 g, Ohaus Analytical Plus) and vacuum distilled water from these samples was collected using a modified version of the pipette method (Nagy, 1983). In brief, frozen flies were transferred into a Pasteur pipette and the blunt end of the pipette was flame sealed. The flies were then immersed in liquid nitrogen and the narrow end of the pipette attached to a vacuum pump to evacuate the air. The pipette was then sealed at the narrow end and placed onto a slide warmer at 50°C, with the narrow end of the pipette projecting out at room temperature. Water distilled from the flies condensed into the narrow end of the pipette, which was gradually moved further off the plate until all the water from the flies was distilled. The narrow end containing the water was then snapped off and resealed. The flies were then reweighed and average water content (*N*) calculated as the difference between the pre- and post-distilled fly weights, divided by the number of flies in the pipette. Samples of the food were treated similarly to measure food water content.

The distillate from the flies and the food was analyzed to determine the isotopic enrichments of ^18^O and ^2^H. For ^18^O, we used the small sample equilibration technique (Speakman et al., 1990), in which a small weighed sample of the water is equilibrated with a known volume of isotopically characterized CO_2_. The resultant CO_2_ was then analyzed using gas source isotope ratio mass spectrometry (IRMS; ISOCHROMμG – Micromass UK Ltd, UK). The ^2^H analysis was performed on hydrogen gas, produced by on-line chromium reduction of water (Morrison et al., 2001). Water samples, contained within 50 μL inserts inside 2 mL septa-sealed vials, were placed on the carousel of a EuroAS 300 liquid autosampler (EuroVector, Italy) fitted with a 1 μL injection syringe (SGE Europe Ltd, UK). Two wash cycles of 1 μL per cycle were done for each sample prior to injection into a chromium chromatographic packed reactor (50 g, 200 μm particle size, Goodfellow, UK) fitted inside a standard EA 3000 elemental analyzer (EuroVector, Italy). Each water sample was sub-sampled five times, and these sub-samples (0.2 μL) were injected into a heated (170–180°C) injector. The resulting water vapor was flushed into the quartz reactor tube (Elemental Microanalysis, UK) by the carrier helium gas, and reduced to hydrogen gas. The H_2_ gas was carried in the helium stream through the gas chromatography column to an open split sampling capillary and into the source of the isotope ratio mass spectrometer (IRMS). We measured the ^2^H:^1^H ratios with a single-inlet IRMS (IsoPrime, Micromass UK Ltd, UK) provided with an electrostatic energy filter that separates the helium tail from the ^1^H^2^H peak (Speakman and Krol, 2005).

Each batch of samples was run adjacent to triplicates of three laboratory standards to correct variation in mass spectrometer performance. All isotope enrichments were measured in δ per mille relative to the working standards and converted to ppm, using the established ratios for these reference materials. Reference standards were characterized every three months relative to SMOW (Standard Mean Ocean Water) and SLAP (Standard Light Antarctic Precipitation) (Craig, 1961) supplied by the International Atomic Energy Agency (Austria). For measurement of isotope enrichment in water samples, five sub-samples of the distilled water for ^2^H and two sub-samples for ^18^O were used. Water derived from the unlabeled food was used to estimate the background isotope enrichment ((Speakman and Racey, 1987) – method B).

Elimination constants for both isotopes (*k*_o_ and *k*_d_ for oxygen and hydrogen, respectively) were calculated using gradients of the log-converted differences between body water enrichments at times *t*, and the background isotope enrichment, plotted as a function of *t*. We converted the values of *k*_o_, *k*_d_ and body water pool size (*N*) to estimates of CO_2_ production using the single pool model (Speakman, 1993). We assumed a fixed evaporation of 25% of the water flux (equation 7.17 (Speakman, 1997)), which has been demonstrated to minimize error in a range of conditions (Visser and Schekkerman, 1999; Van Trigt et al., 2002). CO_2_ production was converted to energy expenditure assuming a respiratory quotient of 0.83.

### Statistics

All values reported are means ± standard error of the mean (SEM), with *P* < 0.05 regarded as statistically significant. In all analyses using general linear modeling (GLM), non-significant interaction effects (*P* > 0.05) were subsequently removed from each analysis in order to obtain the best-fit model. We performed a *post hoc* power analysis to establish the power of our experiments to detect a significant treatment effect on daily energy expenditure.

## Figures and Tables

**Table 1:** Elimination constants for^18^O (*k*_o_) and ^2^H (*k*_d_) from labeled flies.

Subpopulation	*k*_o_ (h^−1^)	*k*_d_ (h^−1^)	*k*_o_/*k*_d_
Virgin females	0.063 ± 0.003	0.055 ± 0.003	1.16
Virgin males	0.065 ± 0.005	0.055 ± 0.004	1.18
Once-mated females	0.067 ± 0.004	0.058 ± 0.004	1.18
Once-mated males	0.062 ± 0.004	0.053 ± 0.003	1.18
Mixed-sex group: females	0.095 ± 0.007	0.084 ± 0.006	1.12
Mixed-sex group: males	0.099 ± 0.011	0.087 ± 0.008	1.14
Mean	0.072 ± 0.003	0.062 ± 0.003	

Data are means ± SEM.
